# Monthly Summary

**Published:** 1880-12

**Authors:** 


### MONTHLY SUMMARY.

*Toxic Effects of Carbolic Acid.*—The toxic effects of carbolic acid are attracting some attention at the present time. In a limited number of cases its contact with the skin in any form is poisonous, producing violent irritation. The probability is that these cases are to be set down as idiosyncrasies, which are liable to present themselves in individual cases here and there in regard to all remedial agents. There are persons whom opium will vomit, others whom it will purge, and everybody knows how many persons exhibit effects from it opposite to the rule. So it is with oil of turpentine, acetate of lead, etc. We have known numbers of individuals who dared not apply to the skin either of the two agents named, as they were certain to produce severe inflammation. Carbolic acid may be placed under the same head. Indeed there is scarcely an article in the materia medica in the use of which the practitioner does not occasionally meet with effects in direct opposition to the regular physiological action.—*Pacific Med. & Surg. Journal.*

*Fatal Carelessness.*—Eddie Hempbell, the son of Charles Hempbel, who resides at No. 6 Hawkins street, died from the effects of an illy extracted tooth as is stated by the attending physician. The facts of the case it seems are these: The little boy had been troubled with the tooth ache and his father took him to a German barber-dentist by the name of Gartman, corner of Franklin and Clinton streets. Gartman had extracted teeth for Mr. Hempbel previously and no trouble had ensued from the operation. After the tooth had been extracted the boy was taken home, but his face commenced swelling almost immediately. The next day Mr. Hempbel summoned Dr. Wilbur, who examined the boy and pronounced an abscess in formation and said that he would lance it the next day. Upon calling the day following the abscess had disappeared, evidently breaking, and the pus absorbed by the blood. The boy from this time began to get worse and Dr. Wilbur called in Dr. Koch for consultation. Their efforts proved of no avail and the boy died, nine days after being sick. The disease is specified among physicians as hospital fever, or a lighter stage of gangrene. The cause is attributed to the unclean condition of the instruments used by the dentist, it being very dangerous at this time of the year to use surgical instruments of any kind after an operation has been performed with them, unless they have been thoroughly cleansed and washed with carbolic acid. —*Sunday Morning Herald.*

---

*Carbolic Acid Poisoning*, according to the *London Medical Record*, may be under two different forms. (1.) Sudden and without apparent cause, the patient sinking under the dressing; five such cases, with one recovery are reported. (2.) Cases in which the septic symptoms are developed only slowly, and after some weeks perhaps. A sudden rise of temperature, it is thought, just after a Carbolic Acid dressing, should be viewed with suspicion. The obvious point remedy is to remove the dressing. Sodic Sulphates, as Glanhu's Salts, seem to form combinations with Carbolic Acid forming sulpho-carbolates, and so perhaps neutralize its effects in the system.

---

*The Goodyear Patent.*—The *Dental Jairus* states that the Goodyear Dental Vulcanite Co. have already made application for a re-issue of their patent, which expires by limitation in June, 1881. We think the *Dental Jairus* is mistaken. The only way in which the Goodyear Dental Vulcanite Co. can procure an extension of the Cummings patent is by special act of Congress, and we are assured that no such application has yet been presented to the Patent Committees of either the Senate or House of Representatives.—*Dental Advertiser.*

